# Sunitinib and Fenofibrate as Combination Therapy for MDR Glioblastoma: Insights from *In Vitro* and *In Silico* Studies

**DOI:** 10.32604/or.2025.073371

**Published:** 2026-03-23

**Authors:** Saad Alobid, Hussam Albassam, Tebyan O. Mirgany, Faris Almutairi, Mohammed Mufadhe Alanazi, Ahmed H. Bakheit, Hanadi H. Asiri, Eram Eltahir, Gamaleldin I. Harisa

**Affiliations:** 1 Department of Pharmacology and Toxicology, College of Pharmacy, King Saud University, Riyadh, 11451, Saudi Arabia; 2 Department of Pharmaceutical Chemistry, College of Pharmacy, King Saud University, Riyadh, 11451, Saudi Arabia; 3 Department of Pharmaceutics, College of Pharmacy, King Saud University, Riyadh, 11451, Saudi Arabia

**Keywords:** Glioblastoma, drug repurposing, mitochondrial membrane potential, reactive oxygen species (ROS), topoisomerase II, matrix metalloproteinase-9, glutathione peroxidase 4

## Abstract

**Objective:**

Glioblastoma (GB) therapy is challenged by tumor heterogeneity and multidrug resistance (MDR), highlighting the need for effective therapies. This study aimed to explore the combined anticancer effects of Sunitinib (SNB) and Fenofibrate (FEN) on U87 cells.

**Methods:**

U87 cells were exposed to SNB, FEN, or their combination for 24 h, followed by evaluations of cell viability, migration, and clonogenic survival using MTT, scratch, and colony formation assays. Intracellular reactive oxygen species (ROS) were quantified via the 2^′^, 7^′^-dichlorofluorescein assay, while mitochondrial membrane potential (MMP) was assessed using JC-1 red/green fluorescence. Molecular docking was performed to investigate SNB and FEN interactions with multiple molecular targets, including topoisomerase II (TOP-II), c-Jun N-terminal kinase (JNK), histone deacetylase 2 (HDAC2), cyclooxygenase-2 (COX-2), matrix metalloproteinase-9 (MMP-9), cytochrome P450 3A4 (CYP3A4), glutathione peroxidase 4 (GPX4), glutathione S-transferase (GST), heme oxygenase-1 (HO-1), and 5-lipoxygenase (5-LOX).

**Results:**

The results demonstrated that both SNB and FEN significantly reduced U87 cell viability, migration, and clonogenic potential, with the combination treatment exhibiting synergistic cytotoxicity. SNB alone markedly increased ROS levels, while FEN, individually or in combination, reduced oxidative stress. Although SNB diminished mitochondrial membrane potential, co-treatment with FEN restored MMP values close to control levels. Docking analyses revealed that SNB displayed strong affinities for TOP-II, JNK, and HDAC2, whereas FEN preferentially interacted with MMP-9, COX-2, CYP3A4, and GPX4, suggesting complementary mechanisms targeting oxidative stress, inflammation, and programmed cell death regulation.

**Conclusion:**

The combination of SNB and FEN represents a promising multi-targeted therapeutic approach against GB. SNB and FEN combination capable of modulating and reprogramming key molecular pathways involved in GB progression and MDR.

## 1 Introduction

Glioblastoma (GB) is the most common and aggressive primary malignant brain tumor in adult males [1]. GB accounts for about 15% of all central nervous system tumors and about 49% of malignant central nervous system cancers [2]. It arises from astrocytes and is characterized by rapid growth and a short time to development [3]. GB is characterized by extensive infiltration and resistance to conventional therapies due to the blood-brain barrier (BBB) and multidrug resistance (MDR) [1–3]. The risk factors of GB include aging, male sex, ethnicity, hereditary factors, head injury, obesity, inflammation, smoking, alcohol consumption, radiation exposure, nitrosamines, xenobiotics, and other risk factors [1,2]. The surplus exposure to GB risk factors induced mutations in the genes related to epidermal growth factor receptor (EGFR), tumor suppression, phosphatase and tensin homolog (PTEN), inflammation, and isocitrate dehydrogenase, resulting in malignant transformation of astrocytes [1,2,4].

GB is associated with the modulation of mTOR signaling, reprogramming [1], and upregulation of the WNT/β-catenin pathway. However, peroxisome proliferator-activated receptors (e.g., PPAR-α) are typically downregulated [5]. As a result of genetic and environmental factors, GB is heterogeneous and presents a multiform [1,2,4]. Moreover, epigenetic modifications of histones involved in DNA methylation and acetylation play a central role in the development and heterogeneity of GB. This modification is attributed to mutations in tumor suppressor genes and amplification of growth-related genes [2]. Therefore, GB cells exhibit profound metabolic changes, including boosted aerobic glycolysis, glucose transporter overexpression, altered lipid metabolism, and glutamine dependency to sustain the Krebs’ cycle activity [1].

Additionally, reactive oxygen species (ROS) play a dual role in tumors, including GB, where they can promote tumor growth and death [6,7]. Excessive levels of ROS enhance cancer cell proliferation and migration, but they can also trigger detrimental effects that result in cell damage [6,7]. Mitochondrial membrane potential (MMP) is vital for energy production and cellular health [8]. A normal MMP indicates healthy mitochondria, while a disruption can affect cellular processes like survival, programmed cell death (PCD), and other processes [8]. In GB, alternation of MMP and mitochondrial dysfunction promotes tumor growth and MDR by altering energy metabolism and ROS production [8]. Moreover, disrupted mitochondrial dynamics further enhance proliferation [9,10]. Targeting mitochondrial aspects and using mitochondria-targeting drugs may improve GB treatment outcomes [8,10].

Indeed, GB cells escape PCD, such as apoptosis, ferroptosis, and other PCD forms [11]. Mutations in genes related to PCD lead to the longevity of GB cells [2]. Which is associated with increased proliferation, migration, invasion, metastasis, and immortality [2]. Specifically, escaping ferroptosis increases GB invasiveness and the development of chemoresistance [4]. Therefore, ample studies suggested ferroptosis as an approach to tumor management [4,11]. Herein, targeting ferroptosis at proteomic, genetic, and epigenetic levels is a promising therapeutic approach for GB management [4]. Additionally, modulation of glucose, lipid, and iron metabolism may enhance the chemosensitivity of GB [4]. Combining ferroptosis modulators, chemotherapy, radiotherapy, immunotherapy, and targeted therapy could improve GB patients’ therapeutic outcomes [4].

It has been reported that the use of medicines such as estrogen, antihistamines, cannabinoids, statins, NSAIDs, and others was inversely associated with GB risk [2]. Moreover, several treatments, including surgery, radiation, and chemotherapy, were developed for GB control [4]. However, GB medicines have limited BBB permeability and high intra-tumoral or inter-tumoral heterogeneity [4]. Therefore, multimodal therapeutic approaches, including combining surgery, radiotherapy, chemotherapy, and supportive care, are recommended for GB management [1,3,12]. Immunotherapies such as chimeric antigen receptor T-cell therapy, vaccination, checkpoint inhibitors, oncolytic viruses, and dendritic cells have also been investigated for GB treatment [1,12]. In addition, nanotechnology was leveraged to localize anticancer drugs at cancer cell spots [1,12,13].

Despite the progress in GB therapeutic approaches, the GB patients’ prognosis is still poor, and there is an urgent need for the discovery of a novel GB therapeutic plan [12]. In this regard, targeted therapies are emerging for controlling GB [1,12,13]. Tyrosine kinase inhibitors (TKIs) block dysregulated EGFR, which drives GB cells’ oncogenesis, proliferation, angiogenesis, and survival. For instance, TKIs members such as sunitinib (SNB) and others competitively bind to the ATP pocket of kinase domains. Herein, they inhibit phosphorylation and downstream growth signaling cascades [14]. Therefore, TKIs could significantly manage several tumors, including kidney, lung, colon, liver, and GB. Yet, GB cells overexpress efflux pumps, mutations in kinase domains, and activation of other compensatory MDR pathways for TKIs [14]. Furthermore, high intratumoral heterogeneity of GB reduces TKIs’ therapeutic impact. Besides, TKIs have low BBB diffusion rates, high doses, and induce systemic toxicities [14]. Also, off-target toxicity arises from the inhibition of kinases. For instance, SNB has multi-targeted TKI inhibition, leading to mitochondrial ROS production. This promotes oxidative stress, triggers cytochrome-C release, and activates pro-apoptotic cascades [15]. Despite these effects on TKIs members, such as SNB and others, TKIs exhibit poor BBB penetration, which limits their accumulation within tumor tissues. Consequently, high dosing is required to reach therapeutic efficacy, which is associated with systemic toxicities and off-target effects [14–16]. Moreover, multiple targets of SNB frequently occur due to the inhibition of non-specific kinases, resulting in unexpected side effects [14–16].

Consequently, combination therapy could resolve therapeutic problems and enhance the therapeutic outcomes of TKIs [14]. Moreover, drug repurposing is suggested as an additional player in the cancer battle [16,17]. In this context, numerous medicines, including antidiabetic, antimicrobial, and other drugs, were repurposed for cancer management, including GB [16,17]. Thus, drug repurposing supposes giving new uses to medicines approved for treating other diseases [18]. For instance, hypolipidemic agents such as statins and others were repurposed as anticancer agents. Specifically, lipid-lowering therapies exhibit pleiotropic actions; however, lipids have ample roles in cellular physiology [19]. Thus, lipid-lowering medicines offer additional health benefits, including anti-inflammatory, improving glucose metabolism, and other health benefits [19]. Several studies documented the beneficial impact of lipid-lowering agents in malignant diseases [19]. PPAR-α agonists exhibit cytotoxic effects against GB [20]. Moreover, these agents promote cell cycle arrest and apoptosis [20]. However, PPAR-α agonists have therapeutic challenges such as BBB penetration variability, GB heterogeneity, and MDR [20].

For instance, fenofibrate (FEN) is converted into the pharmacologically active metabolite fenofibric acid (FFA) that activates the nuclear transcription factor, PPARα agonist [21]. FEN modulates mitochondrial function, dynamics, biogenesis, mitophagy, and energy production [10,20]. FEN exhibits cytotoxic effects against many cancers [20,22]. In addition, FEN inhibited cell proliferation, migration, and angiogenesis [21]. Therefore, FEN exerted antitumor effects in breast, liver, glioma, prostate, pancreas, and lung cancers [22]. Particularly, FEN-induced apoptosis against GB at low systemic toxicity. This suggests that FEN is a good candidate in support of conventional therapies against GB [23]. Despite this, PPAR-α modulators are contradictory in cancer; some studies highlight its anti-tumor potential, while others suggest that PPAR-α activation may promote oncogenesis [24]. These conflicting findings emphasize the need for further research to address the anticancer effect of PPAR-α modulators [24].

Interestingly, the combination of PPAR-α agonists and TKIs represents a promising approach for GB by simultaneously targeting metabolic and proliferative pathways. PPAR-α agonists like FEN modulate lipid metabolism and inflammation, potentially disrupting tumor energy supply and its inflammatory microenvironment. TKIs like SNB block multiple targets in oncogenic signals. This dual strategy may act synergistically, with PPAR-α agonists enhancing TKI efficacy by altering tumor metabolism, MDR, and cancer viability [25]. *In silico* leveraging information from basic, translational research databases and clinical trial studies enables identification of new therapeutic approaches of the existing drugs, and thereby accelerating the drug repurposing process to speed drug discovery while significantly reducing overall development costs [17]. Therefore, using *in silico* studies in drug repurposing speeds drug discovery with reduced costs [17]. Integrating *in silico* studies with laboratory experiments reinforces the therapeutic potential of repurposed drugs [17]. *In silico* studies, artificial intelligence algorithms and bioinformatics tools identify interactions between drugs and protein targets such as receptors or enzymes [17]. Mostly, enzymes are implicated in epigenetic and genetic modulation of metabolism, oncogenic signaling, proliferation, epithelial–mesenchymal transition (EMT), cell colony formation, immune evasion, MDR, and other processes in malignant cells [6,26–28]. Several enzymatic targets play critical roles in GB biology, such as oncogenesis, progression, survival, angiogenesis, metastasis, apoptosis, ferroptosis, MDR, and other PCD types [6,26–28]. Therefore, tumor-associated enzymes represent potential for prediction, diagnosis, monitoring, and therapeutic intervention points for malignant diseases management [6,26–28].

In sum, drug repurposing offers a promising approach to improve GB therapy by combining agents with complementary mechanisms. SNB and FEN may act synergistically to disrupt GB progression. Consequently, this study aims to evaluate the effects of SNB, FEN, and their combination on U87 cells as a GB surrogate model by assessing cell viability, migration, and clonogenic survival after a 24-h treatment compared to control cells. Additionally, it seeks to measure intracellular ROS levels using the 2^′^, 7^′^-dichlorofluorescein (DCF) assay and assess MMP through JC-1 red/green fluorescence analysis. Furthermore, molecular docking analyses were used to explore the multi-target interactions of SNB and FFA. For example, Topoisomerase II (TOP-II), c-Jun N-terminal kinase (JNK), and histone deacetylase 2 (HDAC2), matrix metalloproteinase-9 (MMP-9), cyclooxygenase-2 (COX-2), cytochrome P450 3A4 (CYP3A4), and glutathione peroxidase 4 (GPX4) to investigate their effect on multi-targets involved U87 programming and GB treatment.

## 2 Materials and Methods

### 2.1 Materials

Sunitinib (SNB) (557795-19-4) and Fenofibrate (FEN) (49562-28-9) were purchased from Sigma-Aldrich (St. Louis, MO, USA). All other reagents of the highest commercial grade, unless indicated, were obtained from Sigma Chemical Co. (St. Louis, MO, USA).

### 2.2 Cell Culture

Human glioblastoma astrocytic cells (U87) (ATCC, Manassas, VA, USA). The U87 cell lines used in this study have been Short Tandem Repeat (STR)-certified to ensure their authenticity and genetic stability. U87cells were cultured at 37°C in a humidified incubator with 5% CO_2_ until reaching 70%–80% confluence. When working with U87 (glioblastoma astrocytic cells), ensure sterility by using a laminar flow hood and sterile materials, and regularly test for mycoplasma contamination to maintain cell culture integrity. The cells were maintained in Dulbecco’s Modified Eagle’s Medium (DMEM, 1×; Gibco®®, Grand Island, NY, USA) supplemented with 10% fetal bovine serum (FBS; South American origin, Gibco®®) and 1% penicillin–streptomycin (Thermo Fisher Scientific, 15140148, Waltham, MA, USA). Subculturing was performed when cells reached 85%–90% confluence. Cells from passages 5 to 20, exhibiting 80%–90% confluence, were used for subsequent experiments.

### 2.3 U87 Viability Assay

U87 cells were used as a surrogate model for GB. Viability of U87 cells was assessed using the 3-(4,5-dimethylthiazol-2-yl)-2,5-diphenyltetrazolium bromide (MTT) assay. MTT (298-93-1) was purchased from Sigma-Aldrich Company Ltd. (St. Louis, MO, USA). Cells were seeded in 96-well plates at a density of 2.5 × 10^4^ cells per well in 100 μL of culture medium. The treated groups were classified as follows. Group 1: U87 cells treated with 1% dimethyl sulfoxide (DMSO), which is the control, untreated cells. Group 2: U87 cells treated with SNB (10 μm) dissolved in 1% DMSO. Group 3: U87 cells treated with FEN (100 μm) dissolved in 1% DMSO. Group 4: U87 cells treated with SNB (250 μm) dissolved in 1% DMSO. Group 5: U87 cells treated with FEN (200 μm) dissolved in 1% DMSO. Group 6: U87 cells treated with (FEN 150 μm plus SNB 100 nm), dissolved in 1% dimethyl sulfoxide (DMSO). Treatments were administered for 24 h.

The doses of SNB and FEN were selected depending on the previously published work [29,30]. After treatment for a 24-h duration, 10 μL of the MTT solution (5 mg/mL) was added to each well, and the plates were incubated for 3 h at 37°C in a 5% CO_2_ atmosphere, protected from light. Subsequently, 100 μL of DMSO was added to dissolve the resulting formazan crystals after discarding the medium containing MTT. The plate was then covered with aluminum foil and kept in the dark for 10–15 min. Absorbance was measured at 570 nm using a microplate reader (BioTek, Elx-800, Taunton, MA, USA).

### 2.4 U87 Migration Study

The migratory ability of U87 cells was evaluated using a scratch assay to assess the impact of various treatments on cancer cell migration. Cells were cultured at a seeding density of 3 × 10^5^ cells/per well in 6-well plates until they reached approximately 80% confluence. A linear scratch was created across the cell monolayer using a sterile 200 μL pipette tip. Following scratching, cells were treated for 24 h. Images of the scratched area were captured at 0- and 24-h post-scratch using a 4× objective lens on an EVOS XL Core microscope (Thermo Fisher Scientific, Waltham, MA, USA). The percentage of cell migration was quantified by measuring the number of cells that migrated into the wound area, relative to the control (untreated) group, using ImageJ Version 1.54 g software (NIH, Bethesda, MD, USA).

### 2.5 U87 Colony Formation Study

The clonogenic (or colony formation) assay is an *in vitro* method used to evaluate the ability of a single cell to survive and proliferate into a colony, with a colony defined as a cluster of at least 50 cells. To assess the long-term proliferative capacity of U87 cells and the cytotoxic effects of various treatments, cells were seeded in 12-well plates at a density of 50 cells per well. After allowing the cells to adhere, they were treated with FEN (150 μm), SNB (100 nm), and their combination (FEN 150 μm + SNB 100 nm). The plates were then incubated for 1–3 weeks for colony formation. At the end of the incubation period, the media were gently removed, and the wells were washed with Phosphate Buffered Saline (PBS) solution has a pH (7.4) and a concentration of 137 mm NaCl, 10 mm phosphate, and 2.7 mm KCl. Colonies were stained with 0.5% (w/v) crystal violet, and images were captured using an Apple iPhone 13 Camera. This assay provided insight into the ability of the treatments to inhibit colony-forming potential and long-term cell survival.

### 2.6 Intracellular ROS Production Assessment

Intracellular ROS production was measured using a 96-well plate, where U87 cells were seeded at a density of 3 × 10^4^ cells per well in 100 μL of complete medium and incubated for 24 h. To evaluate whether the combination treatment exerts a protective effect against Sunitinib-induced ROS generation, the H_2_DCFDA Cellular ROS Assay Kit, purchased from MedChem Express (Monmouth Junction, NJ, USA) with a Cat. No.: HY-D0940 was employed. This assay utilizes 2^′^,7^′^–dichlorofluorescin diacetate (H_2_DCFDA), a cell-permeant fluorescent probe that detects hydroxyl, peroxyl, and other reactive oxygen species within cells. Cells were cultured, harvested. Cells were cultured at 37°C in a humidified incubator with 5% CO_2_ until reaching 70%–80% confluence. Media were then aspirated and 0.5 mL of trypsin (0.25% trypsin–0.02% EDTA solution) (Gibco, Thermo Fisher Scientific) was added and incubated for 5 min. Cells were then observed under a microscope to ensure detachment. 4 mL of fresh complete medium was then added, and then pipetted up and down all the adherent cells into a cell suspension. The cell suspension was then transferred into a 15 mL centrifuge tube and centrifuged at 1200 rpm for 5 min. The filtrate was aspirated, and the pellet was resuspended in 2 mL of pre-warmed complete media. After cell counting using a Countess Automated Cell Counter, cells were then seeded at a density of 3 × 10^4^ cells per well in 100 μL of complete medium and incubated for 24 h. ROS assessment was performed after a 1-h pre-treatment with FEN (150 μm), SNB (100 nm), and their combination (FEN 150 μm + SNB 100 nm). Following treatment, H_2_DCFDA (25 μm) was added to each well, and cells were incubated for 30 min. The resulting fluorescence of the oxidized product, DCF, was measured using a microplate reader with excitation and emission wavelengths of 485 and 529 nm, respectively.

### 2.7 Mitochondrial Polarization Study

MMP was assessed as an indicator for mitochondrial polarization using the JC-1 assay in a 96-well plate. U87 cells were seeded at a density of 2 × 10^4^ cells per well and incubated for 24 h to allow for stabilization. The MMP Assay Kit purchased from MedChemExpress (Monmouth Junction, NJ, USA) with a Cat. No.: HY-K0601 (JC-1 dye) was employed to evaluate the impact of various treatments on ∆Ψm. JC-1 is a membrane-permeant dye that accumulates in mitochondria, forming red-fluorescent aggregates (oligomers) in healthy, non-apoptotic cells and remaining as green-fluorescent monomers in depolarized mitochondria. Following the 24-h incubation of the seeding process, cells were treated with FEN (150 μm), SNB (100 nm), and their combination (FEN 150 μm + SNB 100 nm) for 24 h. After treatment, cells were incubated with two μM JC-1 reagent for 15 min at 37°C. Using a fluorescence microplate reader, fluorescence was measured according to the manufacturer’s instructions: red fluorescence (aggregates) with excitation/emission at 530/590 nm and green fluorescence (monomers) at 485/528 nm. The red-to-green fluorescence ratio was calculated for each group to determine mitochondrial membrane potential changes and plotted for analysis.

### 2.8 Molecular Docking Studies

An extensive molecular docking protocol was employed to evaluate the inhibitory capabilities of both synthesized molecules and naturally derived bioactive agents. Molecular docking, a well-established computational tool in pharmaceutical and biochemical research, facilitates the prediction of binding poses and affinities between ligands and target proteins, offering crucial preclinical insights before experimental validation *in vitro* or *in vivo*.

The docking studies used the Molecular Operating Environment (MOE), version 2024. The selected protein targets included topoisomerase II (TOP-II) (PDB ID: 1QZR) [31], c-Jun N-terminal kinase (JNK; PDB ID: 1pmn) [32], histone deacetylase 2 (HDAC2; PDB ID: 4lxz) [33], cyclooxygenase-2 (COX-2; PDB ID: 5ikq) [34], matrix metalloproteinase-9 (MMP-9; PDB ID: 4xct) [35], cytochrome P450 3A4 (CYP3A4; PDB ID: 5ikq) [36], Glutathione Peroxidase 4 (GPX4; PDB ID: 2OBI) [37], Glutathione S-transferase (GST; PDB ID: 18GS) [38], human heme oxygenase-1 (hHO-1; PDB ID: 3HOK) [39], and Stable-5-Lipoxygenase (5-LOX; PDB ID: 6N2W) [40].

Ligands were first optimized through energy minimization, partial charge assignment, and protonation at physiological pH (7.4) using MOE’s Protonate 3D module [41]. These structures were stored in MOE’s database (MDB) format for subsequent use [42–45]. Protein structures were similarly prepared by adding hydrogen atoms, optimizing receptor geometries, and minimizing energy to ensure structural stability. Binding sites were defined using the coordinates of co-crystallized ligands to target biologically relevant pockets.

Docking simulations were parameterized with the MMFF94x force field. Ligand placement utilized the Triangle Matcher algorithm, with initial scoring performed using the London dG function. For each ligand, 30 conformers were generated and subsequently refined using the Induced Fit method, followed by rescoring with the GBVI/WSA dG scoring function [46,47].

Post-docking evaluations focused on analyzing ligand binding conformations, hydrogen bond interactions, and steric complementarity within the active sites. Hydrogen bonds were considered meaningful when donor–acceptor distances were within 3.5 Å. This approach enabled a detailed and predictive assessment of the compounds’ binding affinities and interaction patterns, shedding light on their potential inhibition mechanisms.

### 2.9 Statistical Analysis

Data were analyzed using GraphPad InStat software, version 5 (GraphPad, ISI Software Inc., La Jolla, CA, USA). A one-way analysis of variance was employed to compare the results. Data are presented as mean ± SD, with a *p*-value of <0.05 indicating statistical significance.

## 3 Results

### 3.1 Influence of SNB and FEN and Their Combination on U87 Viability

**Figure 1:**
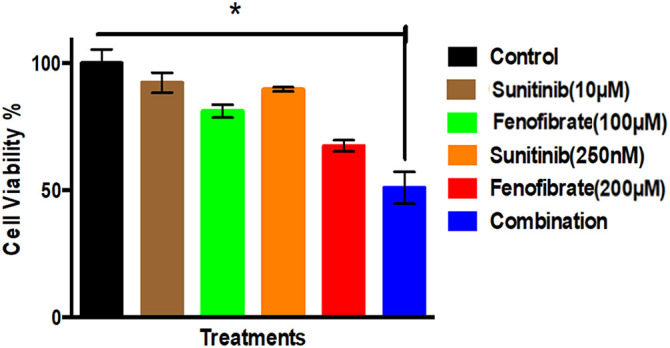
Effect of different doses of SNB and FEN or their combination treatment on U87 viability. The data are expressed as the mean ± SD, six samples per group, and marks indicate the presence of a significant increase or decrease from the control. *, significant decrease, at *p* value < 0.05

Fig. 1 compares the percentage of cell viability across different treatment conditions with the untreated control. The results indicated that the control groups maintained high cell viability, while the individual drug treatments of SNB and FEN showed a reduction in cell viability. The most pronounced decrease in cell viability appears to be in the combination group, suggesting a potential synergistic effect where the two drugs, when combined, are more effective at reducing viable cells than either drug alone.

### 3.2 Influence of SNB and FEN and Their Combination on Wound Healing

**Figure 2:**
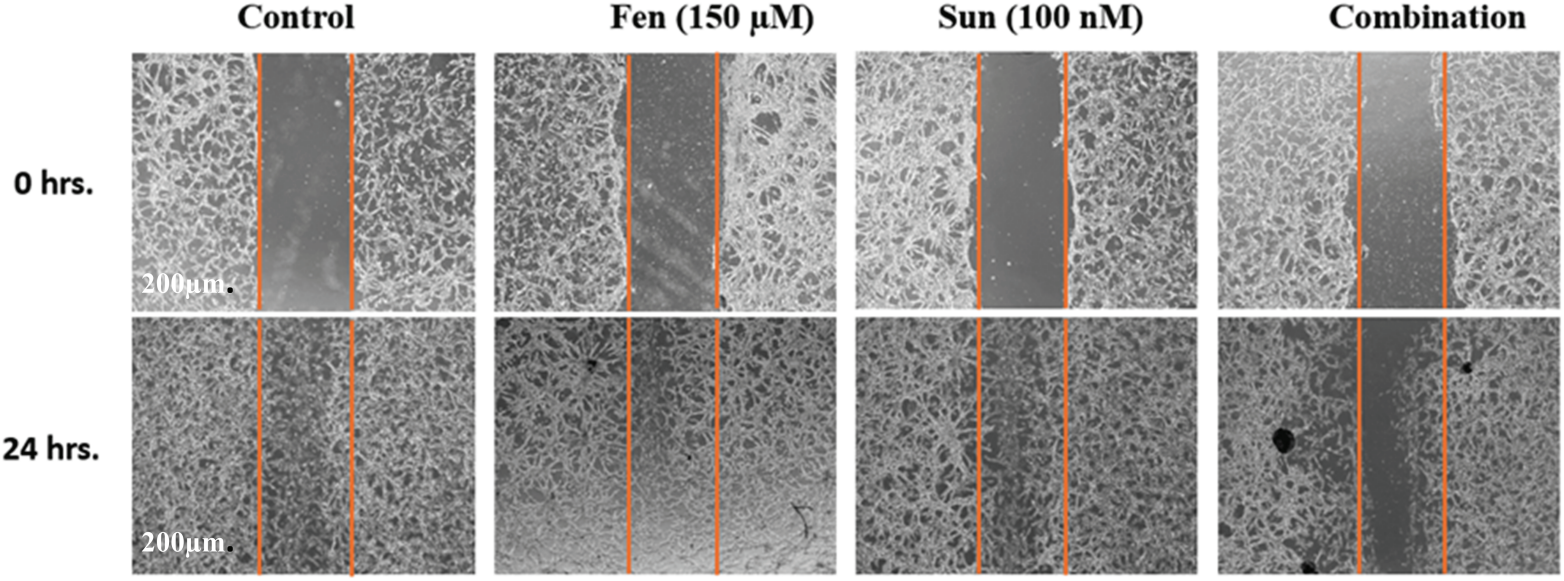
Representative micrographs of wound healing assay showing the effects of SNB and FEN, and their combination on U87 cell migration at 0 and 24 h. Orange dashed lines indicate wound edges. The combination treatment markedly inhibited wound closure compared to single treatments and control. Scale bar = 200 μm

Fig. 2 displays illustrative images from a wound healing assay assessing the effects of SNB and FEN, as well as their combination, on cell migration at 0 and 24 h. At 0 h, all groups display a clear wound gap, outlined by orange dashed lines. After 24 h, the control group shows nearly complete closure. SNB or FEN treatments partially inhibited migration, whereas the combination of SNB and FEN treatment produced the most significant inhibition, maintaining the widest wound gap. These findings suggest that the SNB and FEN combined treatment synergistically suppresses cell migration.

### 3.3 Influence of SNB and FEN and Their Combination on U87 Cells Migration

**Figure 3:**
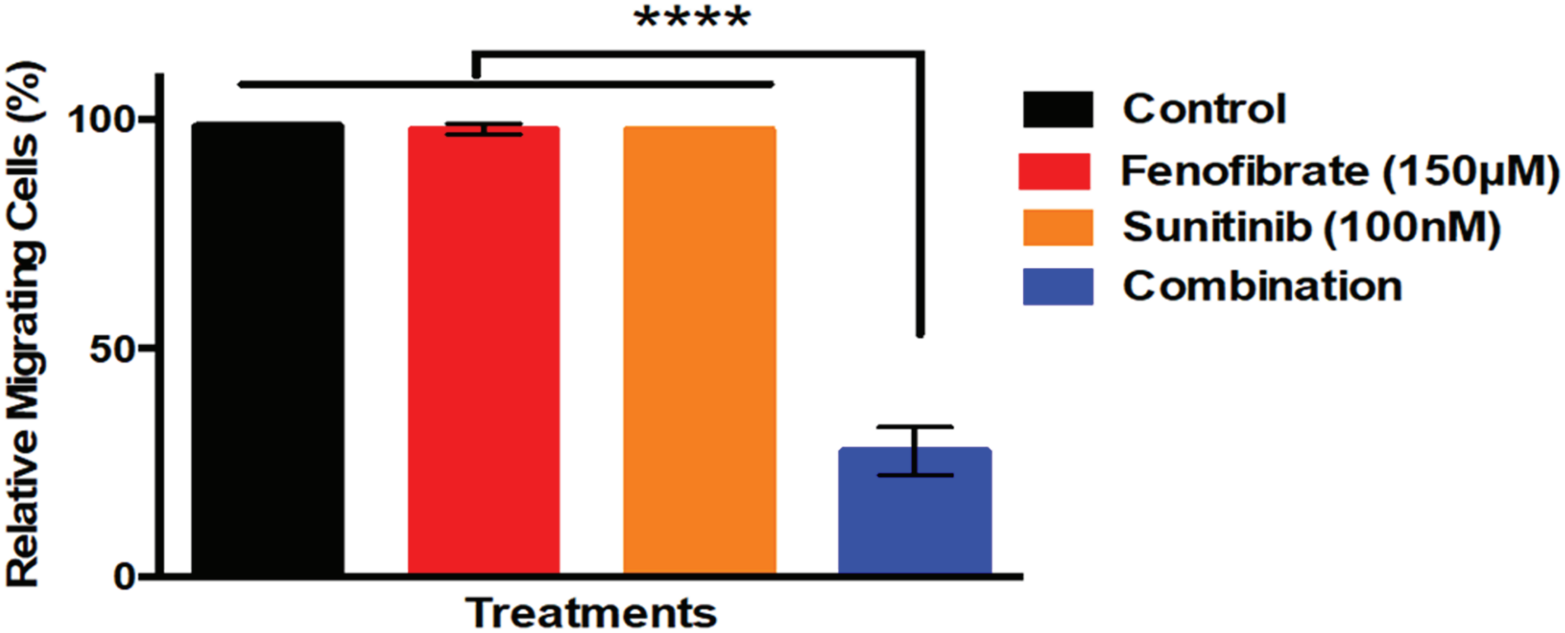
Effect of different doses of SNB and FEN or their combination treatment on U87 migration. The data are expressed as the mean ± SD, six samples per group, and marks indicate the presence of a significant increase or decrease from the control. ****, significant decrease, at *p* value < 0.0001

Fig. 3 presents the percentage of U87 cells migration under different treatment conditions. The control, SNB, FEN, and SNB plus FEN groups exhibit comparable migration levels, with values close to 100%, indicating minimal inhibition of cell movement. In contrast, the combination treatment of SNB and FEN markedly reduces cell migration to approximately 25% of the control level. The statistical analysis reveals a significant difference between the SNB and FEN combination group and all other treatment groups. These results confirm that the combination of SNB and FEN exerts a strong synergistic inhibitory effect on cell migration.

### 3.4 Influence of SNB and FEN and Their Combination on Colony Formation

**Figure 4:**
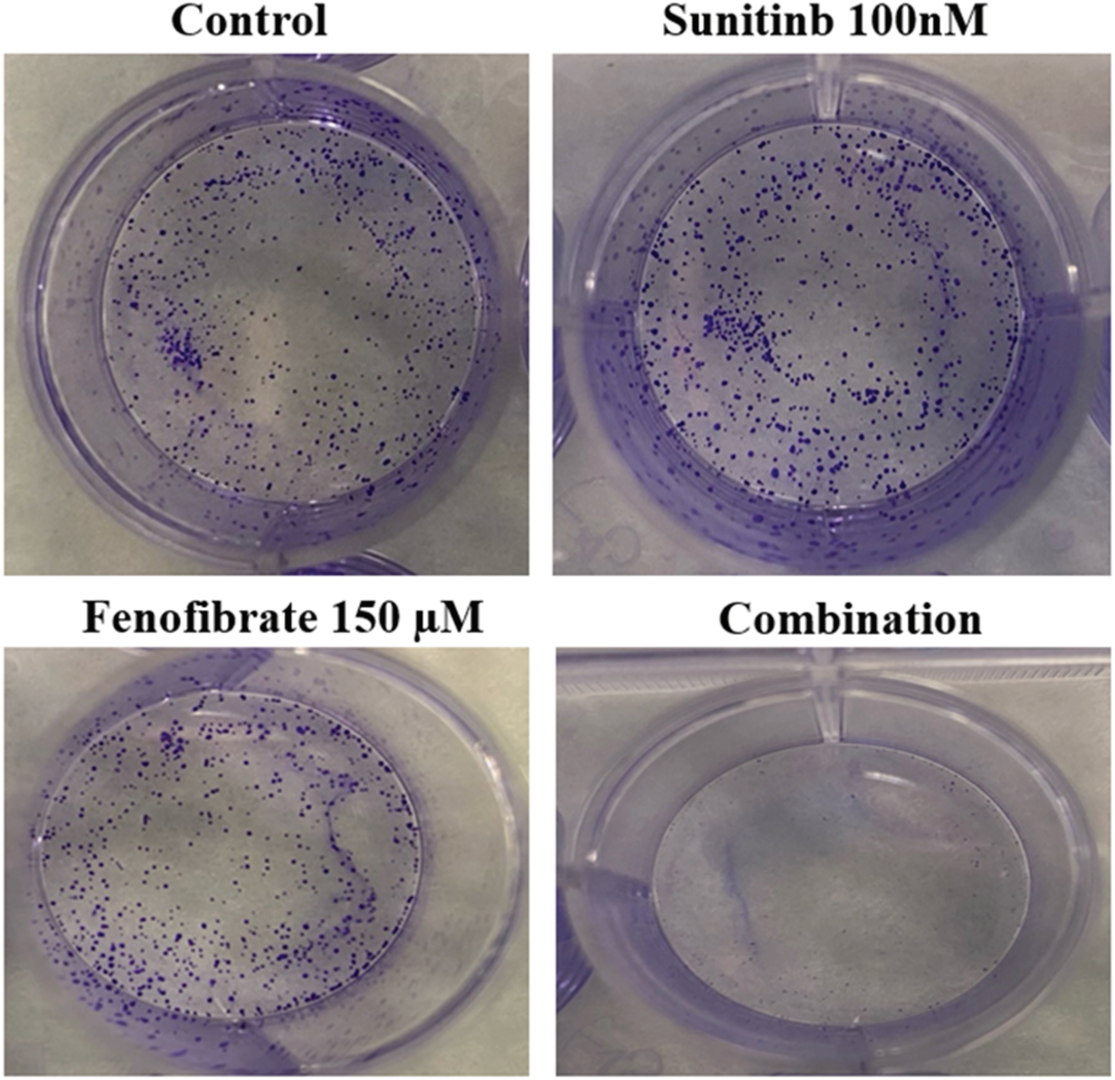
Effect of different doses of SNB and FEN or their combination treatment on U87 colony formation

Fig. 4 shows representative images from a clonogenic assay demonstrating the effects of SNB and FEN, as well as their combination, on cell colony formation. The SNB-treated groups display a high number of dense, stained colonies, indicating robust cell proliferation and survival. The FEN-treated group shows a moderate reduction in colony number, suggesting partial inhibition of cell growth. In contrast, the combination treatment of SNB and FEN markedly reduces colony formation, with only a few faint colonies visible, indicating a strong synergistic suppression of clonogenic potential.

### 3.5 Influence of SNB and FEN and Their Combination on ROS Production

**Figure 5:**
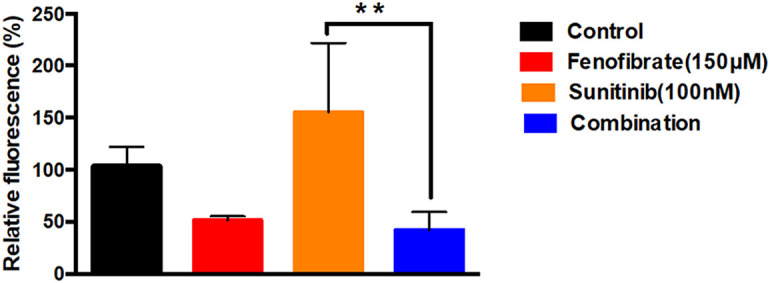
Effect of different doses of SNB, FEN, or their combination treatment on U87 on ROS production. The data are expressed as the mean ± SD, six samples per group, and marks indicate the presence of a significant increase or decrease from the control. **, significant decrease, at *p* value < 0.01

Fig. 5 shows the effect of different doses of SNB, FEN, or their combination on relative fluorescence as an indicator for ROS production. The control group displays baseline fluorescence at approximately 100%, while FEN reduces fluorescence to around 50%. Treatment with SNB markedly increases fluorescence, indicating a strong enhancing effect. In contrast, the combination of SNB and FEN dramatically decreases fluorescence to roughly 40%, effectively counteracting the increase induced by SNB alone. FEN substantially suppresses the fluorescence elevation caused by SNB.

### 3.6 Influence of SNB and FEN and Their Combination on MMP

**Figure 6:**
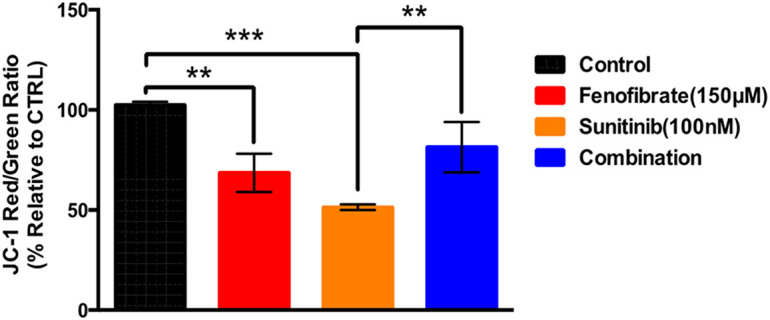
Effect of different doses of sunitinib and FEN or their combination treatment on U87 on mitochondrial potential. The data are expressed as the mean ± SD, six samples per group, and marks indicate the presence of a significant increase or decrease from the control. **, significant decrease, at *p* value < 0.01, ***, significant decrease at *p* value < 0.001

Fig. 6 illustrates the effect of SNB, FEN, or their combination on the JC-1 red/green fluorescence ratio, expressed as a percentage relative to the control. The control group exhibits a normalized JC-1 ratio of approximately 100%, while FEN and SNB both significantly decrease this ratio to about 70% and 50%, respectively, indicating mitochondrial membrane depolarization. In contrast, the SNB plus FEN treatment partially restores the JC-1 ratio to roughly 80%, suggesting a protective or compensatory effect when both drugs are used together. SNB induces a strong mitochondrial effect that is mitigated by co-treatment with FEN co-therapy.

### 3.7 Molecular Docking

Molecular docking, a well-established computational tool in pharmaceutical and biochemical research, facilitates the prediction of binding poses and affinities between ligands and target proteins, offering crucial preclinical insights before experimental validation *in vitro* or *in vivo*. In the present study, an extensive molecular docking protocol was employed to evaluate the modulatory capabilities of both SNB and FEN against multiple targets (TOP-II, JNK, HDAC2, COX2, MMP-9, CYP3A4, GPX4, GST, HO-1, and 5-LOX). These enzymes are interconnected in pathways that regulate cell development, survival, and metastasis in cancer, highlighting their potential as therapeutic targets. Table 1 represents molecular docking results for FFA and SNB with TOP-II, JNK, HDAC2, COX2, and MMP-9 enzymatic targets. As well, Table 2 indicates molecular docking results for FFA and SNB with CYP 3A4, GPX4, GST HO-1, and 5-LOX enzymatic targets. Both Tables 1 and 2 demonstrate the key ligand–receptor interactions, bond distances (Å), estimated binding energies, and docking scores (kcal/mol).

**Table 1:** Summary of molecular docking results for FFA and SNB with TOP-II, JNK, HDAC2, COX2, and MMP-9 enzymatic targets. The table lists key ligand–receptor interactions, bond distances (Å), estimated binding energies, and docking scores (kcal/mol)

Enzymes	Ligand			Receptor			Interaction	Distance	E (kcal/mol)	Score (kcal/mol)
TOP-II	FFA	O	1	ND2	ASN	142 (B)	H-acceptor	3.03	−0.4	−8.07
		C	35	6-ring	TYR	28 (A)	H-pi	4.12	−0.3	
	SNB	N	29	OE1	GLN	365 (B)	H-donor	2.8	−5.3	−8.35
		O	51	CA	GLN	365 (B)	H-acceptor	3.2	−0.6	
		6-ring		N	THR	27 (A)	pi-H	4.39	−0.3	
JNK	FFA	C	17	SD	MET	146 (A)	H-donor	4.27	−0.3	−7.58
		C	25	SD	MET	146 (A)	H-donor	3.71	−0.5	
		6-ring		CG1	VAL	196 (A)	pi-H	3.63	−0.6	
	SNB	N	29	SD	MET	146 (A)	H-donor	3.34	−3.8	−8.46
		5-ring		CG1	VAL	78 (A)	pi-H	4.38	−0.6	
		6-ring		CB	LEU	206 (A)	pi-H	4.83	−0.3	
HDAC2	FFA	O	1	NE2	HIS	146 (A)	H-acceptor	2.8	−1.5	−6.74
		O	1	ZN	ZN	401 (A)	Metal	1.94	−1.3	
		C	39	6-ring	PHE	210 (A)	H-pi	4.1	−0.7	
		6-ring		6-ring	PHE	155 (A)	pi-pi	4.06	−0.2	
	SNB	N	29	O	GLY	154 (A)	H-donor	2.94	−0.7	−6.43
		C	45	O	GLY	143 (A)	H-donor	3.11	−0.3	
		O	51	NE2	HIS	146 (A)	H-acceptor	2.72	−2.9	
		O	51	ZN	ZN	401 (A)	Metal	1.92	−1.7	
		5-ring		6-ring	PHE	155 (A)	pi-pi	3.83	−0.2	
COX-2	FFA	6-ring		CA	SER	353 (A)	pi-H	3.44	−0.4	−8.22
		6-ring		CB	ALA	527 (A)	pi-H	3.58	−0.4	
	SNB	O	51	OG	SER	530 (A)	H-acceptor	2.86	−1	−7.41
		6-ring		CA	GLY	526 (A)	pi-H	4.23	−0.7	
		5-ring		CA	ALA	527 (A)	pi-H	3.9	−0.5	
MMP-9	FFA	O	1	CA	LEU	187 (A)	H-acceptor	3.53	−0.3	−7.54
	SNB	C	19	O	LEU	243 (A)	H-donor	3.41	−0.3	−7.75
		N	29	OE2	GLU	227 (A)	H-donor	2.94	−5.2	
		O	51	O	HOH	517 (A)	H-acceptor	2.89	−1.9	
		5-ring		CA	LEU	187 (A)	pi-H	3.97	−0.5	
		6-ring		CD1	LEU	187 (A)	pi-H	3.66	−0.3	
		5-ring		N	LEU	188 (A)	pi-H	4.35	−0.5	

Note: TOP-II, topoisomerase II; JNK, c-Jun N-terminal kinase; HDAC2, histone deacetylase 2; COX-2, cyclooxygenase-2; MMP9, matrix metalloproteinase-9; SNB, sunitinib; FFA, fenofibric acid.

**Table 2:** Summary of molecular docking results for FFA and SNB with CYP 3A4, GPX4, GST, HO-1, and 5-LOX enzymatic targets. The table lists key ligand–receptor interactions, bond distances (Å), estimated binding energies, and docking scores (kcal/mol)

Enzymes	Ligand			Receptor			Interaction	Distance	E (kcal/mol)	Score (kcal/mol)
CYP 3A4	FFA	O	23	O	HOH	2024 (A)	H-acceptor	2.89	−0.8	−7.78
	SNB	C	53	5-ring	HEM	1501 (A)	H-pi	3.92	−0.3	−7.93
		5-ring		CG2	THR	309 (A)	pi-H	3.87	−0.9	
GPX4	FFA	C	25	OD2	ASP	21 (A)	H-donor	3.35	−0.3	−6.47
	SNB	N	30	OH	TYR	63 (A)	H-donor	3.26	−0.5	−3.77
		N	37	O	HOH	306 (A)	H-donor	3.25	−1.1	
		S	8	CA	TYR	63 (A)	H-acceptor	4.34	−0.5	
		S	8	CD1	TYR	63 (A)	H-acceptor	4.12	−0.3	
GST	FFA	CL	9	OE2	GLU	97 (B)	H-donor	3.69	−0.5	−6.19
		CL	9	SG	CYS	101 (B)	H-donor	3.47	−0.5	
		O	1	OG	SER	65 (A)	H-acceptor	3.05	−0.8	
		C	31	5-ring	TRP	38 (A)	H-pi	4.69	−0.4	
	SNB	O	25	OH	TYR	7 (A)	H-acceptor	3.06	−2.7	−7.06
		N	29	6-ring	TYR	108 (A)	H-pi	3.85	−0.5	
HO-1	FFA	6-ring		CA	GLY	143 (B)	pi-H	4.65	−0.3	−7.36
		6-ring		5-ring	HEM	300 (B)	pi-pi	4.23	−0.2	
		6-ring		5-ring	HEM	300 (B)	pi-pi	4.33	−0.1	
		6-ring		6-ring	PHE	214 (B)	pi-pi	4.09	−0.2	
	SNB	O	51	CA	ASP	140 (B)	H-acceptor	3.37	−0.4	−7.65
		C	32	5-ring	HEM	300 (B)	H-pi	4.5	−0.8	
		5-ring		5-ring	HEM	300 (B)	pi-pi	3.96	−0.2	
5-LOX	FFA	O	23	OG1	THR	364 (B)	H-acceptor	3.09	−0.3	−6.46
		C	4	5-ring	HIS	372 (B)	H-pi	3.99	−0.3	
		6-ring		CB	LEU	368 (B)	pi-H	5.02	−0.3	
	SNB	N	22	OE1	GLN	363 (B)	H-donor	3.2	−0.5	−7.10
		N	48	OD1	ASN	407 (B)	H-donor	3.51	−0.3	
		5-ring		CA	ASN	407 (B)	pi-H	4.55	−0.7	

Note: CYP3A4, cytochrome P450 3A4; GPX4, glutathione peroxidase 4; GST, glutathione S-transferase; HO-1, heme oxygenase-1; 5-LOX, 5-lipoxygenase; SNB, sunitinib; FFA, fenofibric acid.

**Figure 7:**
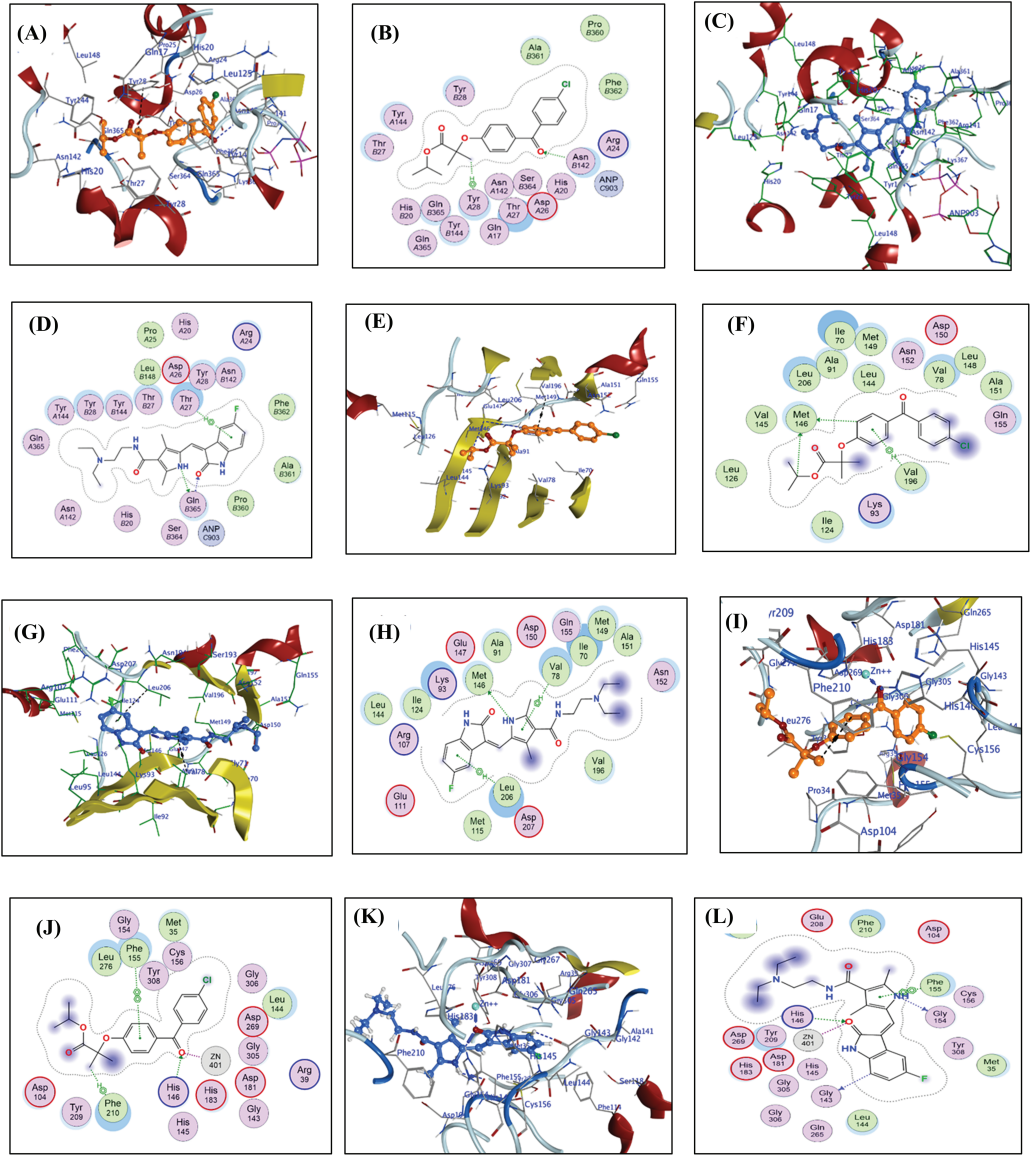
Docking poses of FFA and SNB bound to target enzymes. Panels (**A**–**L**) illustrate 3D (left subpanel) and 2D (right subpanel) complexes: TOP-II (panels (**A**–**D**)), JNK (panels (**E**–**H**)), and HDAC2 (panels (**I**–**L**)). FFA and SNB are represented as orange and blue ball-and-stick models, respectively, with key target residues labeled

**Figure 8:**
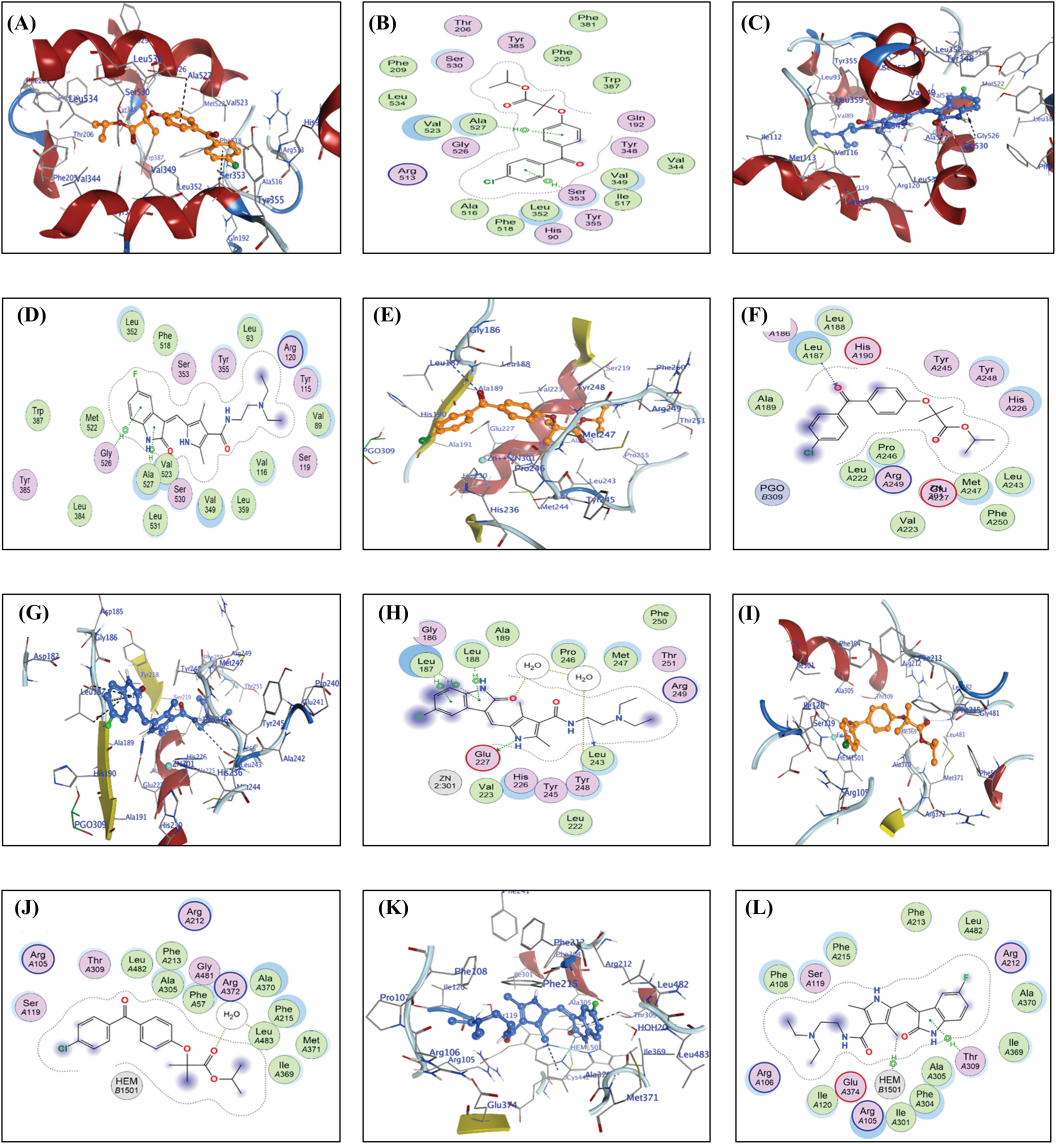
Docking poses of FFA and SNB bound to target enzymes. Panels (**A**–**L**) illustrate 3D (left subpanel) and 2D (right subpanel) complexes: COX-2 (panels (**A**–**D**)), MMP-9 (panels (**E**–**H**)), and CYP3A4 (panels (**I**–**L**)). FFA and SNB are represented as orange and blue ball-and-stick models, respectively, with key target residues labeled

**Figure 9:**
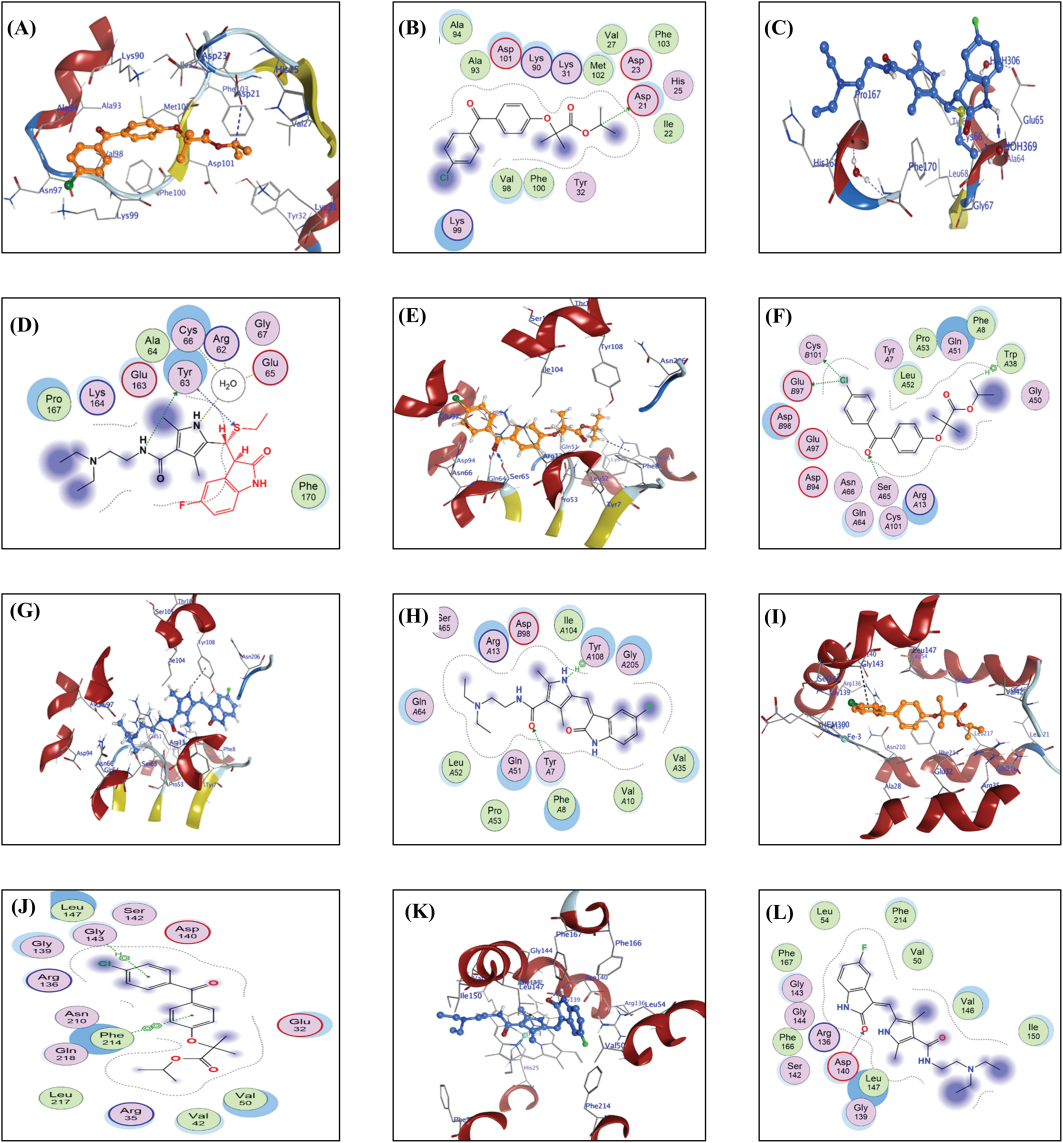
Docking poses of FFA and SNB bound to target enzymes. Panels (**A**–**L**) illustrate 3D (left subpanel) and 2D (right subpanel) complexes GPX4 (panels (**A**–**D**)), GST (panels (**E**–**H**)), and HO-1 (panels (**I**–**L**)). FFA and SNB are represented as orange and blue ball-and-stick models, respectively, with key target residues labeled

**Figure 10:**
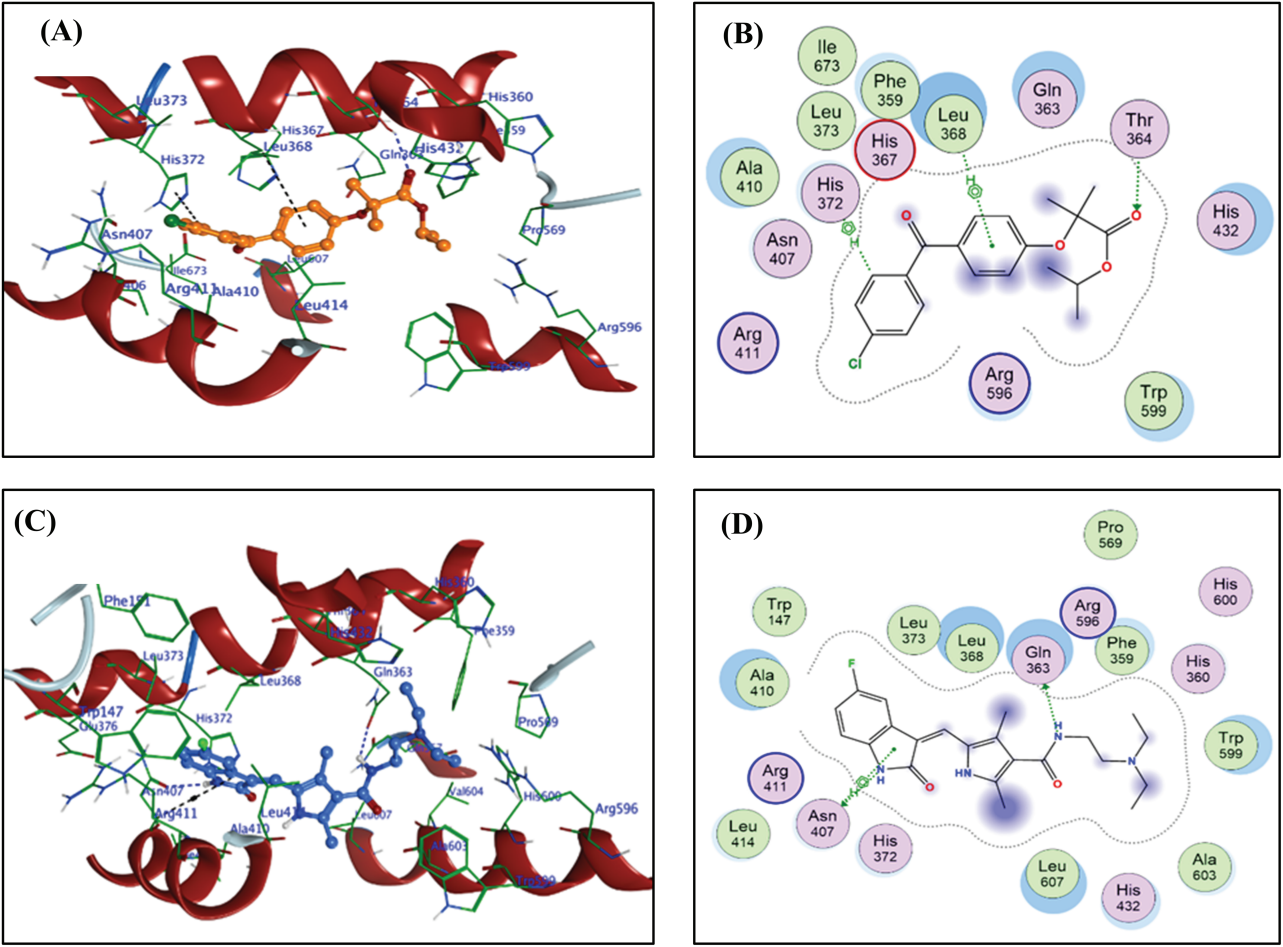
Docking poses of FFA and SNB bound to target enzymes. Panels (**A**–**D**) illustrate 3D (left subpanel) and 2D (right subpanel) complexes. 5-LOX (panels (**A**–**D**)). FFA and SNB are represented as orange and blue ball-and-stick models, respectively, with key target residues labelled

For each FFA and SNB complex enzyme complex, a 3D structural view displays the ligand’s binding orientation within the enzyme’s active site. At the same time, a corresponding 2D interaction diagram details the specific molecular forces stabilizing the complexes. These include conventional hydrogen bonds and potential ionic interactions, depicted as dashed lines, which often involve key interactions, hydrogen bonds, π–π stacking, and metal coordination with Zn^2+^ are indicated by dashed lines. FFA and SNB are depicted as orange and blue ball-and-stick models, respectively, with key target residues labeled. Fig. 7 shows TOP-II, JNK, and HDAC2 interaction with FFA and SNB. Fig. 8 indicates COX2, MMP-9, and CYP3A4 interaction with FFA and SNB are represented as orange and blue ball-and-stick models, respectively, with key target residues labeled. Fig. 9 displays FFA and SNB binding to GPX4, GST and HO-1. Moreover, Fig. 10 reveals FFA and SNB binding to 5-LOX.

## 4 Discussion

The drug combination therapy achieves high efficacy with reduced side effects by using lower doses to target tumor subpopulations resistant to single agents [48]. However, some cancer cells are entering dormancy, later reactivating to drive relapse, often years after therapy [48]. Dormant cells and cancer stem cells are key drivers of GB recurrence and MDR, posing significant challenges to treatment effectiveness [48]. The discovery of new therapeutic plans, particularly targeted small-molecule agents, has expanded the scope of combination treatment strategies [49]. Thus, the modulation of glucose, lipid, and iron metabolism may increase the chemosensitivity and reprogramming of GB [4]. The drug combination approaches could improve the therapeutic outcomes of GB patients [1]. However, this approach simultaneously targets multiple oncogenic pathways, such as metabolism, PCD, cyclin-dependent kinases, tumor suppressors, and growth factors [50]. Moreover, combination therapies often outperform monotherapies by providing synergistic effects and overcoming tumor heterogeneity [50]. For instance, combining a PPAR-α agonist with TKIs sensitizes cancer cells to targeted therapy. Thus, a combination of PPAR-α agonist with chemotherapy, hormonotherapy, targeted therapy, and immunotherapy has facilitated the incorporation of PPAR-α agonist into cancer treatment strategies [51].

Therefore, this study was conducted to explore the combined use of SNB and FEN as a novel multitarget approach for treating GB. Their potential synergistic effects on U87 as a GB surrogate model were investigated by evaluating cell viability, migration, and proliferation. Additionally, the study measured reactive ROS levels to assess oxidative stress and analyzed MMP to understand apoptotic processes. Molecular docking analyses were also performed to characterize the interactions between SNB and FEN with specific molecular targets and to elucidate the underlying molecular mechanisms involved in this interaction. Overall, this study provided insights into the therapeutic benefits of combining SNB and FEN, laying the groundwork for future clinical applications in GB treatment.

### 4.1 U87 Viability

In the present study, FEN, SNB and their combination exerted significant U87 cell death. The FEN and SNB combination therapy shows a more substantial inhibitory impact, indicating a synergistic effect. These findings are concurrent with several studies that suggest the cytotoxic effect of these medicines. SNB is a multi-targeted TKI [52], which blocks many growth factor receptor kinases overexpressed or amplified in GB. Thus, SNB exhibited a significant cytotoxic effect on U87 cells [52]. Moreover, FEN exhibits cytotoxic effects on U87 cells [20]. However, PPAR-α agonists induce metabolic reprogramming, leading to energy deprivation in tumor cells [20]. Moreover, these agents promote cell cycle arrest, apoptosis, and suppress pro-survival signals [20]. Similarly, FEN inhibited tumor growth at the epigenetic level [53]. It has been reported that PPAR-α agonists modulate metabolism, inflammation, immune response, and adjust proliferation, differentiation, and apoptosis of cancer cells [54]. Particularly, FEN regulates cell cycle progression, induces apoptosis, and inhibits cell proliferation and EMT by activating PPAR-α [53]. These actions suppress DNA methyltransferase 1 (DNMT1) activity, reverse cyclin-dependent kinase inhibitor 2A (CDKN2A) methylation, and restore CDKN2A expression [53]. This induces cell cycle arrest, thereby inducing cell cycle arrest will be induced via the CDKN2A/retinoblastoma protein (RB)/E2F transcription factor pathway [53]. This study confirmed the anticancer potential of FEN and its reduction of tumor growth at the epigenetic level [53].

### 4.2 U87 Migration

The EMT is a critical process in cancer metastasis, enabling epithelial cells to lose adhesion and acquire migratory and invasive characteristics that facilitate their departure from the primary tumor site. EMT allows cancer cells to partially lose epithelial traits while gaining mesenchymal features, resulting in increased motility and the potential for metastasis [55]. In the current investigation, treating U87 cells with SNB, FEN, or their combination reduced the migration capability of U87 cells. The drug combination produced a pronounced effect compared to free drugs. Similarly, several studies indicated that TKIs disrupt pathways that promote cancer cell migration [56,57]. Specifically, SNB, a multi-targeted TKI, significantly inhibits cancer cell migration and metastasis [56,57]. This action disrupts critical signaling pathways related to angiogenesis and tumor growth, diminishing the invasive potential of cancer cells [58]. It has been indicated that SNB can lower the migratory potential in ample cancer cells [56]. Additionally, SNB modulates several signaling pathways and reduces the activity of matrix metalloproteinases such as MMP-2 and MMP-9, which are linked to cell migration [56,57], and SNB has also been shown to inhibit the EMT [58]. Overall, SNB and other TKIs offer a promising approach to counteracting cancer metastasis by inhibiting signals that facilitate cell migration and invasion [56,57].

Similarly, several studies have reported that PPAR-α agonists, such as FEN and others, suppress the growth of GB cells by inhibiting cell proliferation, migration, and angiogenesis, as well as by inducing the apoptotic pathway [20,59]. FEN also interferes with metabolic pathways and inflammatory signaling, potentially enhancing the sensitivity of cancer cells [20,59]. In sum, the combination of SNB and FEN could lead to novel interventions that inhibit GB cell migration. Targeting multiple pathways may enhance therapeutic efficacy and improve treatment outcomes for patients with GB.

### 4.3 U87 Colony Formation

In the present study, the SNB and FEN combination significantly affected colony formation in the U87 cell line. SNB, a multi-targeted TKI, disrupts critical signaling pathways involved in U87 colony formation by inhibiting cell viability and the EMT. These actions of SNB diminish the invasive potential of GB cells and modulate the tumor microenvironment through angiogenesis inhibition [56–58]. Additionally, FEN suppresses GB cell proliferation and induces apoptosis, thereby limiting colony formation [20,59]. Moreover, FEN alters metabolic pathways, impacting the energy dynamics of cancer cells [20,59]. SNB and FEN represent promising therapeutic strategies for managing GB growth, colony formation, and metastasis. Their combined effects may enhance treatment efficacy and improve patient outcomes in GB management.

### 4.4 Intracellular ROS Production

ROS can enhance cancer cell proliferation and migration; excessive ROS can induce oxidative stress, leading to cell death [6,7]. TKIs disrupt signaling pathways and potentially modulate ROS production. FEN improves mitochondrial function and decreases fatty acid synthesis, reducing ROS production. Moreover, FEN could induce apoptosis in GB cells through oxidative stress [20]. A previous study reported that controlling ROS levels is a potential strategy for GB treatment [6]. In the current work, the DCF assay further revealed an increase in ROS upon treatment of U87 with SNB. However, there is a substantial reduction in intracellular ROS levels following FEN or SNB plus FEN treatment.

It has been reported that SNB disrupts several signaling pathways [15], which promotes oxidative stress, triggers cytochrome-C release, and activates pro-apoptotic cascades [15]. On the contrary, FEN attenuates, enhancing the antioxidant defense system in cancer cells [60]. Furthermore, FEN modulates oxidative stress responses [61]. PPAR-α agonists can protect against chemical-induced neurodegeneration [62]. It has been demonstrated that FEN is a potential medication to treat diet-induced neurodegenerative disorders [63]. Likewise, FEN mitigates neuropathology by modulating oxidative stress, autophagy, mitochondrial dysfunction, and inflammatory-signaling pathways [64]. FEN activation of PPARα promotes the β-oxidation of fatty acids and inhibits fatty acid synthesis [21]. Lipoprotein-associated phospholipase A2, pro-inflammatory cytokines, and oxidative stress [21]. A previous study established FEN as an inhibitor of cancer cell proliferation through modulation of apoptosis and mitochondrial function [29]. SNB and FEN can modulate oxidative stress in GB. SNB induces oxidative stress, increases ROS production to promote apoptosis and inhibits the proliferation of cancer cells. While primarily for lipid regulation, FEN also affects metabolic pathways and ROS levels. Their combination may enhance ROS reprogramming, leading to enhanced therapeutic outcomes by inhibiting GB growth and metastasis.

### 4.5 MMP

MMP and mitochondrial function are crucial for cellular health and energy metabolism. A high MMP indicates healthy mitochondria that support ATP production, while a decrease can signal dysfunction, affecting cellular processes such as apoptosis [8]. In cancer cells, altered MMP and mitochondrial dysfunction can promote survival and resistance to therapies. MMP can be assessed with fluorescent dyes, evaluating mitochondrial health in various contexts, including cancer. MMP, mitochondrial function, and dynamics influence tumor survival and death [8]. In GB, mitochondrial dysfunction often leads to altered energy metabolism and increased ROS production, promoting tumor progression. Mitochondrial dynamics, characterized by fission and fusion, are frequently disrupted in GB cells, enhancing their proliferation and resistance to therapies [8]. Mitochondrial dynamics, biogenesis, and mitophagy about GB proliferation, besides mitochondria-targeting drugs, are suggested for GB therapy [9]. Targeting these mitochondrial aspects may enhance the effectiveness of anticancer treatments [8].

In the present work, compared to the control group, both FEN and SNB administered individually significantly reduced MMP, with SNB demonstrating a more substantial decrease. Notably, when FEN was used in conjunction with SNB, there was a significant improvement in the JC-1 ratio compared to treatment with SNB alone. These finding indicates that the combination therapy effectively mitigates the adverse effects of SNB on mitochondrial function, suggesting a partial restoration of MMP when both agents are used together. Similarly, it has been reported that SNB induces mitochondrial dysfunction by inhibiting survival signaling pathways, elevation the ROS, and altering the MMP [14–16]. The disruption of mitochondrial function with SNB treatment reflects enhanced induction of mitochondrial-mediated apoptosis [15]. However, SNB is a multi-targeted TKI that inhibits adenosine monophosphate-activated protein kinase (AMPK), leading to mitochondrial ROS generation, energy deprivation, apoptosis, and cell death [15]. Additionally, SNB directly inhibits ample growth factor receptors and disrupts the Raf-MEK-ERK and PI3K-Akt-mTOR signaling pathways, which results in oxidative stress and mitochondrial dysfunction [15]. Therefore, SNB reduces tumor-induced neurodegeneration and inhibits tumor progression [14–16]. Conversely, FEN improves mitochondrial bioenergetics by promoting fatty acid oxidation, enhancing mitochondrial biogenesis, stabilizing MMP, and reducing ROS. This modulation can lead to decreased tumor growth and improved therapeutic outcomes. However, FEN-activated AMPK [21], which contributed to the anti-apoptotic effects of FEN [21]. It has been reported that the anti-apoptotic effect of FEN is independent of PPARα activity [21]. The activation of AMPK by FEN also increased nitric oxide production and reduced expression of adhesion molecule genes [21]. In sum, the combination of SNB and FEN offers a promising treatment for GB by inducing MMP to enhance efficacy and reduce MDR. Thus, targeting MMP presents a promising strategy for GB treatment.

### 4.6 Molecular Docking Analyses

In the present study, the selected protein targets are TOP-II [31], JNK [32], HDAC2 [33], COX-2 [34], MMP9 [35], CYP3A4 [36], GPX4 [37], GST [38], HO-1 [39], and 5-LOX [40]. Therapeutically, touching these enzymes offers diverse opportunities to reprogram cancer cells. In this context, targeting JNK or HDAC2 can restore cell growth, apoptosis, ferroptosis, and tumor suppressor activity, while inhibiting COX-2 or 5-LOX may suppress inflammation-driven tumor growth. Inactivation of MMP-9 reduces invasiveness, and inhibition of GPX4 or HO-1 induces oxidative stress or ferroptosis. Together, targeting these enzymes supports multi-targeted therapeutic strategies of the FEN and SNB combination.

In the present work, *in silico* studies in terms of the molecular docking analyses of SNB and FFA active metabolites of FEN against 10 enzymatic targets revealed distinct binding profiles that illuminate their mechanisms of action and support their classification as inhibitors rather than substrates [65]. SNB has been reported as a therapeutic agent in the treatment of several cancers [65]. TOP-II inhibitors are indicated in the therapy of GB [66]. Docking simulations were performed using the Molecular Operating Environment (MOE, v2024). TOP-II is a crucial enzyme involved in DNA management during replication and repair, and it is often overexpressed in GB, contributing to the tumor’s aggressive nature. This enzyme is essential for cancer cell proliferation as it alleviates torsional stress during DNA processes [67]. In the topoisomerase II active site, SNB (blue) nestles more deeply into the nucleotide binding groove than FFA (orange), forging two strong hydrogen bonds with GLN365 (2.8 and 3.2 Å) and a π–H contact with THR27 that effectively occludes substrate entry, whereas FFA makes a single weaker H-acceptor interaction with ASN142 (3.03 Å) and a π–H contact to TYR28. TOP-II inhibitors are used in cancer treatment and are being explored for GB due to their ability to induce apoptosis [67].

Concerning the kinase family that modulate, cell growth, proliferation and apoptosis [68], SNB’s engagement of JNK exemplifies classical type I inhibition; it donates a 3.34 Å H-bond to the hinge region MET146. It establishes dual π–H contacts with VAL78 and LEU206, stabilizing the DFG-out conformation that precludes ATP coordination [69]. FFA, by contrast, interacts with JNK primarily through distal contacts—two H-donor interactions to MET146 (3.71–4.27 Å) and one π–H to VAL196, yielding a slightly weaker affinity (−7.58 kcal/mol) and suggesting sub-maximal inhibition rather than productive phosphorylation turnover [70].

Epigenetic modulators of ferroptosis exert cytotoxic effects by targeting HDACs, encouraging ferroptosis in fibrosarcoma cells, and preventing ferroptosis in neurons. This suggests HDAC inhibitors may induce ferroptosis in GB without causing neurotoxicity [4,71]. SNB is an epigenetic modulator, underscored by its coordination to Zn^2+^ and hydrogen bonding within HDAC2: the metal chelation distance of 1.92 Å and an H-bond to HIS146 (2.72 Å) mimic the hydroxamate warhead of classical inhibitors, thereby blocking substrate access to the catalytic tunnel [72]. FFA also chelates Zn^2+^ (1.94 Å) and hydrogen bonds to HIS146 (2.8 Å), albeit with lower overall binding energy (−6.74 kcal/mol), consistent with a weaker but still inhibitory interaction that could modulate histone deacetylation [73].

COX-2 is overexpressed in many types of cancers exerting a pleiotropic role in promotion of oncogenesis and MDR to therapy [74]. In the preset study, FFA exhibits notable selectivity: in COX-2, it forms two π–H interactions with SER353 and ALA527 (3.44–3.58 Å), replicating known NSAID binding modes to block arachidonic acid access (−8.22 kcal/mol) SNB’s COX-2 binding (−7.41 kcal/mol) is less pronounced and relies on a single H-acceptor to SER530 (2.86 Å) and π–H stacking, suggesting comparatively weaker inhibition.

SNB’s strong π–H stacking to the heme moiety in CYP3A4 (3.92 Å) and FFA’s water-mediated H-bond to a catalytic water molecule (2.89 Å), both preclude substrate oxidation, implicating both compounds as metabolic inhibitors rather than alternative substrates [36]. SNB’s covalent-mode docking with GPX 4 and FFA’s carboxylate engagement with ASP21 (3.35 Å) in GPX 4 reinforce an inhibitory mechanism that precludes peroxide reduction. However, GPX has substantial roles in oncogenesis at different levels [75].

Considering GST, HO-1, and 5-LOX, neither ligand assumes a catalytic alignment; instead, both occupy the active pockets in orientations that sterically block substrate binding and coordinate essential cofactors or residues [76]. In GST, SNB forms a strong H-acceptor interaction with TYR7 (3.06 Å), whereas FFA’s dual contacts to GLU97 and CYS101 (3.47–3.69 Å) mimic substrate orientation but lock the enzyme in a nonproductive state.

The docking orientations, interaction networks, and binding energies presented SNB and FFA as inhibitors of their respective targets, not substrates. SNB operates as a multi-target kinase and epigenetic inhibitor with high potency and diverse binding modes [77]. At the same time, FFA delivers more focused inhibition against inflammatory and metabolic enzymes, aligning with its clinical anti-inflammatory and lipid-modulating roles [78].

In sum, molecular docking analyses of SNB and FFA suggest their potential as multi-target inhibitors with complementary mechanisms. SNB shows higher binding affinities, especially within kinase and epigenetic enzyme families, and effectively inhibits JNK through strong hydrogen bonding, blocking ATP binding with downstream kinases and cell signaling. Its ability to chelate Zn^2+^ and mimic hydroxamate binding in HDAC2 indicates its role in epigenetic modulation and oncogenic gene regulation. In contrast, FFA exhibits a more selective inhibitory profile, particularly for inflammatory and metabolic enzymes like COX-2, 5-LOX and CYP3A4, suggesting a strong anti-inflammatory effect alongside its PPAR-γ agonist activity. FFA’s interactions with antioxidant enzymes also indicate its role in modulating oxidative stress, which is crucial for cell survival. Despite SNB’s stronger affinities, FFA’s selective engagement with metabolic and inflammatory targets supports its use in combination therapies. Both compounds dock in orientations adopt docking orientations that inhibit activity across various targets, reinforcing their classification as inhibitors. This multi-target approach is significant for GB treatment, addressing tumor heterogeneity, MDR, and treatment failures.

The novelty of this study lies in exploring FEN, a lipid-lowering drug, as a repurposed adjuvant therapy in combination with SNB against U87, GB cells. This dual-drug strategy integrates anti-angiogenic metabolic programming and targeting multiple signaling pathways simultaneously. Overall, this study introduces a novel pharmacological combination with the dual advantage of repurposing an FDA-approved drug (FEN and SNB) and enhancing therapeutic selectivity and safety, representing a potential step toward overcoming MDR in GB.

The main limitations of this study include its reliance on a single cell line (U87) and *in vitro* experiments, which limit the generalizability of the findings. Moreover, the short treatment duration may not capture long-term cellular effects. The predicted molecular interactions from docking were not experimentally validated. Additionally, toxicity and selectivity toward normal cells were not assessed, and the absence of quantitative synergy analysis, such as combination index values, reduces the precision of evaluating drug interactions. Further *in vivo* and mechanistic studies are planned to confirm these results and assess clinical relevance.

## 5 Conclusion

The present study concludes that the combination of SNB and FEN showcases significant therapeutic potential against GB by concomitantly hindering the proliferation, migration, and clonogenic survival of U87 cells. While SNB exerts strong anti-proliferative effects through multi-targeted kinase and epigenetic inhibition, FEN enhances this effect by reducing oxidative stress, modulating metabolic pathways, and partially restoring MMP. Molecular docking analyses confirmed the complementary binding profiles of SNB and FEN, targeting multi-targets (TOP-II, JNK, HDAC2, COX2, MMP-9, CYP3A4, GPX4, GST, HO-1, and 5-LOX) that are essential proliferative, inflammatory, and metabolic enzymes involved in tumor development and MDR. This multi-pronged approach addresses tumor heterogeneity and MDR, positioning it as an effective adjuvant therapy that may enhance treatment efficacy for GB patients. Collectively, these findings suggest that repurposing SNB and FEN, whether used individually or in combination, represents a promising multi-targeted treatment strategy for reprogramming of cancer cells and management of GB. Further preclinical and clinical studies are necessary to validate and translate these findings into practical clinical applications.

## Data Availability

All data generated or analyzed during this study are included in this published article.
